# Technological Interventions for Medication Adherence in Adult Mental Health and Substance Use Disorders: A Systematic Review

**DOI:** 10.2196/12493

**Published:** 2019-03-12

**Authors:** Jackson M Steinkamp, Nathaniel Goldblatt, Jacob T Borodovsky, Amy LaVertu, Ian M Kronish, Lisa A Marsch, Zev Schuman-Olivier

**Affiliations:** 1 Boston University School of Medicine Boston, MA United States; 2 Outpatient Addiction Services Department of Psychiatry Cambridge Health Alliance Somerville, MA United States; 3 Washington University School of Medicine St Louis, MO United States; 4 Tufts University School of Medicine Boston, MA United States; 5 Center for Behavioral Cardiovascular Health Columbia University Irving Medical Center New York City, NY United States; 6 Center for Technology and Behavioral Health Geisel School of Medicine at Dartmouth Lebanon, NH United States; 7 Department of Psychiatry Harvard Medical School Boston, MA United States

**Keywords:** systematic review, mental health, substance-related disorders, mHealth, psychiatry, medication adherence, medication compliance

## Abstract

**Background:**

Medication adherence is critical to the effectiveness of psychopharmacologic therapy. Psychiatric disorders present special adherence considerations, notably an altered capacity for decision making and the increased street value of controlled substances. A wide range of interventions designed to improve adherence in mental health and substance use disorders have been studied; recently, many have incorporated information technology (eg, mobile phone apps, electronic pill dispensers, and telehealth). Many intervention components have been studied across different disorders. Furthermore, many interventions incorporate multiple components, making it difficult to evaluate the effect of individual components in isolation.

**Objective:**

The aim of this study was to conduct a systematic scoping review to develop a literature-driven, transdiagnostic taxonomic framework of technology-based medication adherence intervention and measurement components used in mental health and substance use disorders.

**Methods:**

This review was conducted based on a published protocol (PROSPERO: CRD42018067902) in accordance with the Preferred Reporting Items for Systematic Reviews and Meta-Analyses systematic review guidelines. We searched 7 electronic databases: MEDLINE, EMBASE, PsycINFO, the Cochrane Central Register of Controlled Trials, Web of Science, Engineering Village, and ClinicalTrials.gov from January 2000 to September 2018. Overall, 2 reviewers independently conducted title and abstract screens, full-text screens, and data extraction. We included all studies that evaluate populations or individuals with a mental health or substance use disorder and contain at least 1 technology-delivered component (eg, website, mobile phone app, biosensor, or algorithm) designed to improve medication adherence or the measurement thereof. Given the wide variety of studied interventions, populations, and outcomes, we did not conduct a risk of bias assessment or quantitative meta-analysis. We developed a taxonomic framework for intervention classification and applied it to multicomponent interventions across mental health disorders.

**Results:**

The initial search identified 21,749 results; after screening, 127 included studies remained (Cohen kappa: 0.8, 95% CI 0.72-0.87). Major intervention component categories include reminders, support messages, social support engagement, care team contact capabilities, data feedback, psychoeducation, adherence-based psychotherapy, remote care delivery, secure medication storage, and contingency management. Adherence measurement components include self-reports, remote direct visualization, fully automated computer vision algorithms, biosensors, smart pill bottles, ingestible sensors, pill counts, and utilization measures. Intervention modalities include short messaging service, mobile phone apps, websites, and interactive voice response. We provide graphical representations of intervention component categories and an element-wise breakdown of multicomponent interventions.

**Conclusions:**

Many technology-based medication adherence and monitoring interventions have been studied across psychiatric disease contexts. Interventions that are useful in one psychiatric disorder may be useful in other disorders, and further research is necessary to elucidate the specific effects of individual intervention components. Our framework is directly developed from the substance use disorder and mental health treatment literature and allows for transdiagnostic comparisons and an organized conceptual mapping of interventions.

## Introduction

### Background

Medication adherence—the set of behaviors relevant to taking one’s medication as directed—is critical to the effectiveness of pharmacologic therapies and improvement of patient outcomes. Psychiatric and substance use disorders are no exception to this rule; studies have repeatedly demonstrated the association between different measures of medication adherence and a wide variety of psychiatric outcomes. Medication nonadherence among individuals with schizophrenia has been shown to be associated with violence [1], hospital admission [2], and mortality [3]. Among those with mood disorders (eg, major depressive disorder and bipolar disorder), nonadherence is associated with hospitalizations, suicide risk, and slower initial recovery [4,5]. Nonadherence can be particularly problematic when treating those with opioid use disorders, as it is associated with treatment dropout [6], which is in turn associated with continued use of illicit drugs [7] and increased mortality [8]. Such studies strongly suggest the important role played by medication adherence across psychiatric disorders. The prevalence of nonadherence in these disorders is also quite high; studies estimate that 41% to 50% of patients with schizophrenia [9], 13% to 52% of those with depression [10], 57% of those with anxiety [11], and 68% of those with opioid use disorder [12] are not fully adherent to their medication. Outside of clinical practice, nonadherence and inaccurate adherence measurement stymie research studies of new interventions; clinical trials must recruit more participants, spend more money, and may even produce erroneous results because of nonadherence [13,14].

Clinicians have been struggling to solve the medication adherence problem for decades; the body of research conducted across a wide variety of medical disorders is large and continues to grow rapidly [15]. The etiologies of nonadherence are complex and multifactorial; perhaps the most common framework includes 5 major categories—socioeconomic factors, health system–related factors, condition-related factors, treatment-related factors, and patient-related factors [16]. In psychiatry, the Perceptions and Practicalities Approach classifies modifiable risk factors of nonadherence into a “ *perceptual* category (relating to patient beliefs, eg, about the presence and severity of their disorder, medication efficacy, and side effect profile) and a *practical* category (eg, resources necessary to acquire medication and cognitive capacity to adhere despite adequate motivation)—suggesting 2 broad targets for intervention [17]. Somewhat parallel to this is the distinction between *voluntary* nonadherence (eg, due to lack of insight or desire) and *involuntary* nonadherence (eg, due to cognitive limitations, poor understanding, or simply forgetting) [18].

Mental health and substance use disorders present unique challenges to adherence owing to the nature of the symptoms associated with these disorders. The delusional belief systems and poor insight often found in schizophrenia appear to be associated with lower levels of adherence [9,19]. The street value of opioid partial agonists (the cornerstone of modern office-based opioid treatment for opioid use disorders) raises the specter of medication diversion and provides a strong incentive for nonadherence. Patients with posttraumatic stress disorder (PTSD), in particular PTSD induced by *medical* events, are more likely to be nonadherent to medication regimens—it has been proposed that the medications may serve as aversive reminders that trigger avoidant behaviors [20]. Depressed patients are much less likely to adhere to medication regimens [21], likely mediated through a decreased motivation for self-care [22]. At least one study has linked anxiety disorders to increased medication nonadherence [23], though other studies have found mixed results [24]. Across all psychiatric disorders, the self-stigma associated with receiving treatment for mental illness has also been shown to contribute to nonadherence [25]. In particular, these challenges may motivate an increased emphasis on adherence *monitoring* in addition to adherence motivation, relative to nonpsychiatric disorders.

In the past few decades, information technology and related fields have enabled a wide variety of new approaches and modalities to improve medication adherence and adherence monitoring. The near-ubiquity of mobile phones [26], the relative ease of app development, the boom in Internet of Things devices capable of recording and transmitting real-time biological data, and the steady improvement of artificial intelligence (AI) and machine learning systems have all contributed to this increase in technology-based interventions for medication adherence.

The field of technological interventions for medication adherence in psychiatric disorders is quite young and expanding in scope, with multiple new trials each year in each disorder context. Many intervention types have been studied only in pilot studies, or in randomized controlled trials (RCTs) with small sample sizes. Furthermore, many interventions consist of multiple components used in conjunction, which complicates the study of the efficacy of individual intervention components. Significant additional research needs to be done before we can determine which technology-based interventions are most promising.

Recent related research in summarizing the vast and diverse field of psychiatric medication adherence technologies includes systematic reviews focusing on particular disorders such as mood disorders [27], schizophrenia and other psychotic disorders [28,29], and substance use disorders [30]. Other reviews evaluate specific technologic intervention types, including automated reminders [31], adherence monitoring [32], and virtual reality technologies [33]. However, given the breadth, size, and expansion rate of this field, little research has been done to compare and contrast feasibility and effectiveness of various interventions *across* disorder contexts, or to provide a transdiagnostic framework of adherence intervention components. Although the efficacy of specific interventions may ultimately prove to differ between diagnoses, we believe that the etiologies of nonadherence and the frequently studied intervention components are similar enough to justify such a transdiagnostic approach. In this review, we also evaluate this assumption formally.

### Objective

Here, we conduct a systematic scoping review of the literature concerning technology-based solutions for medication nonadherence among those with psychiatric disorders. The review is designed to provide a broad transdiagnostic overview of research themes and intervention types, with an eye toward developing a data-driven conceptual framework of technology-based adherence interventions for mental health and substance use disorders. We believe that such a framework would aid researchers in evaluating new literature, create promising directions for future research, and provide a summary of this vast field for the busy clinician who may be overwhelmed by the panoply of available options. In the long term, new multicomponent interventions might be built quickly and effectively by sampling promising components from this framework, based on domain-specific knowledge from a researcher or clinician’s area of study.

## Methods

### Overview

This is a systematic scoping review of technological interventions designed to improve medication adherence in mental health and substance use populations. We define technology broadly, including software, hardware, algorithms, and biosensors. This review was conducted based on a published protocol (PROSPERO: CRD42018067902). We conducted a qualitative synthesis of themes in the included literature, constructed graphical concept maps and graphed topic area popularity over time in different psychiatric disorder contexts. We aimed to provide a comprehensive transdiagnostic framework with which to think about and categorize components of technological interventions for medication adherence in mental health and substance use populations.

### Search Strategy

To obtain relevant scientific literature, we searched 7 electronic bibliographic databases: MEDLINE, EMBASE, PsycINFO, the Cochrane Central Register of Controlled Trials (CENTRAL), Web of Science, Engineering Village, and ClinicalTrials.gov. Study records identified from ClinicalTrials.gov were used to search for related published articles and conference proceedings. Reference lists from the included studies as well as reviews on related topics were searched for additional relevant articles. Authors were contacted to answer specific questions when necessary.

Using the Population-Intervention-Comparison-Outcome (PICO) framework [34], 3 core thematic concepts were used to build the search strategy: mental health and substance use disorders (population), technology (intervention), and medication adherence or compliance (outcome); all 3 of which were necessary for article inclusion. We constructed the final search strategy through an iterative collaborative process, which included reading related review articles, building a tree of relevant MeSH terms, and testing searches against a growing corpus of ”must-include“ articles identified through a preliminary search. Furthermore, we consulted with multiple research librarians to ensure our strategy was comprehensive and database-specific.

We searched by title or keyword, using MeSH terms for MEDLINE and analogous terms for other databases when applicable (eg, Emtree). We initially included English language articles published between January 2000 and January 2018; articles from before 2000 were unlikely to be relevant from a technological perspective. Reviews, letters, case reports, editorials, and other forms of nonprimary research were excluded. Journal articles, conference proceedings, and abstracts were included if they represented primary research. Given the broad scope and heterogeneity of the topic, we included all study designs, ranging from RCTs to pilot feasibility studies to provide a comprehensive concept map. For completeness, a search for any updated manuscripts was rerun on MEDLINE, EMBASE, PsycINFO, CENTRAL, and Web of Science during September 2018. The full search strategies are listed in Multimedia Appendix 1.

### Inclusion Criteria

#### Adherence

Much of the literature around technological adherence interventions relates to improving the accuracy of adherence *measurement* (eg, ingestible biosensors, smart pill bottles, and directly observed remote medication ingestion). Accurate adherence measurement is critical for evaluating the efficacy of interventions both in clinical practice and in research studies, it and is a critical part of many multicomponent interventions. We therefore considered a study to meet the *adherence* criteria of inclusion if the intervention was designed to improve either medication adherence or the accuracy of adherence measurement. We excluded articles which evaluated participant adherence to the intervention as a whole, or a component of the intervention other than adherence to pharmacologic therapy (for instance, a study evaluating whether participants were adherent to a Web-based psychoeducational program). Articles that did not directly measure medication adherence or medication adherence measurement accuracy as an outcome (eg, pilot feasibility or usability studies) were included if medication adherence was a key motivating factor for development of the intervention, as assessed by the abstract, introduction, and discussion sections of the article.

#### Technology

Given the aim of the scoping review, we defined technology as broadly as possible. We wanted to include all forms of modern information technology such as mobile phones, mobile apps, websites, and computer vision-based (CV) AI systems, as well as interactive voice response (IVR) technology. Furthermore, physical technology such as smart pill bottles and ingestible sensors, as well as studies of novel biomarkers or biological measurement methods, were considered to meet the *technology* criterion for inclusion. Many of the multicomponent interventions found throughout the course of the search contained some technological components while also containing other components that did not meet our definition of technology-based; we included these studies and will describe the technological components therein to provide the most comprehensive map of the space.

#### Other Exclusions

We excluded articles that did not exclusively study a psychiatric or substance use population (although we did include articles in which all the participants had an additional nonpsychiatric condition—eg, patients with HIV and opioid use disorder). We excluded epidemiologic studies that only examined the prevalence of nonadherence. We excluded studies which provided only financial incentive for medication adherence with no other components. We excluded protocol-only articles if outcome articles were available for the same study. We excluded articles focusing on pediatric populations, as their caretakers are often responsible for their adherence behaviors. Similarly, we excluded studies of patients with dementia disorders, as they often have caretakers who are responsible for adherence behaviors.

#### Study Selection and Extraction

We used the Cochrane Covidence tool [35] to assist in conducting most of the systematic review. To be included, an article had to be deemed relevant at 2 stages—1) title and abstract screen and 2) full-text screen. Overall, 2 reviewers (JMS and NG) independently screened each abstract and full-text article. Conflicts were resolved via discussion until consensus was reached; a third reviewer (JB) was on hand to resolve disagreements, but this was not needed for this review. Interrater reliability was calculated for both the title-abstract stage and the full-text screening stage using Cohen kappa.

Included articles were then extracted using a charting tool developed iteratively by the authors. Both reviewers (JS and NG) performed extraction on each of the articles and compared the extracted results. Extracted data fields included the following: author’s name, year of publication, study objectives (primarily targeted at improving adherence, measurement accuracy, or both), population (eg, opioid use disorder or bipolar disorder), study type (eg, RCT or pilot feasibility), study size, components of intervention delivered technologically (eg, reminders, education, support, or monitoring), and technological modality when applicable (eg, app, website, or IVR). We did not assess risk of bias or study quality given the large amount of heterogeneity in study populations, study designs, intervention types, and outcome measures as well as the goal to conduct a systematic scoping review.

Before starting the review, we chose to make a top-level distinction between *data collection components* (system components designed to collect data or improve the accuracy of adherence measurement) and *interventional components* (system components designed to directly increase medication adherence).

Results were labeled as including a data collection component if the study either (1) evaluated the accuracy of a measurement method (for instance, a study which compared clinician ratings of adherence to electronic pill monitors) or (2) evaluated an interventional system which periodically assessed participant adherence as *part of the intervention*, not merely a *study endpoint* (eg, a mobile phone app which notifies clinicians in real time of nonadherence events and encourages them to intervene would count as a ”data collection” intervention, but an RCT which evaluated the effects of a psychoeducational program on adherence using electronic pill monitors merely as an *endpoint* would not fall under this category).

#### Synthesis

For this scoping review, we performed a qualitative synthesis of the included studies. We categorized the studies by study type, intervention type, population studied, and technological modality. We created a hierarchical taxonomy of intervention components.

## Results

### Study Selection and Extraction

**Figure 1:**
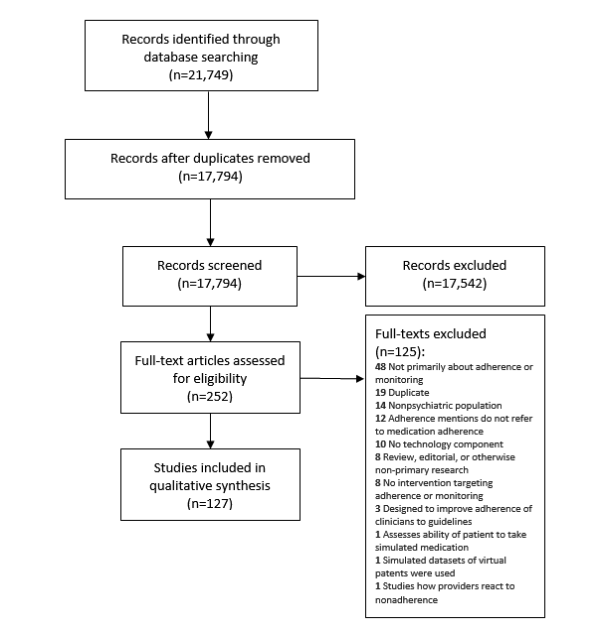
Preferred Reporting Items for Systematic Reviews and Meta-Analyses Diagram of included studies.

The literature search identified 21,749 results. A search of related reviews and references in relevant articles did not find any additional results. Of these, 3955 duplicates were removed, and 17,542 results were excluded based on the title and abstract, yielding 252 results (Cohen kappa before reconciliation=0.82, 95% CI 0.78-0.86). These were screened based on their full texts, and 125 were excluded (Cohen kappa before reconciliation=0.80, 95% CI 0.72-0.87). This left a total of 127 articles and conference proceedings that met the inclusion criteria (Figure 1). In the final rerun of the search in September 2018, 3 indexed manuscripts were found that replaced conference proceedings describing the same studies. All included studies are provided in Multimedia Appendix 2.

**Figure 2:**
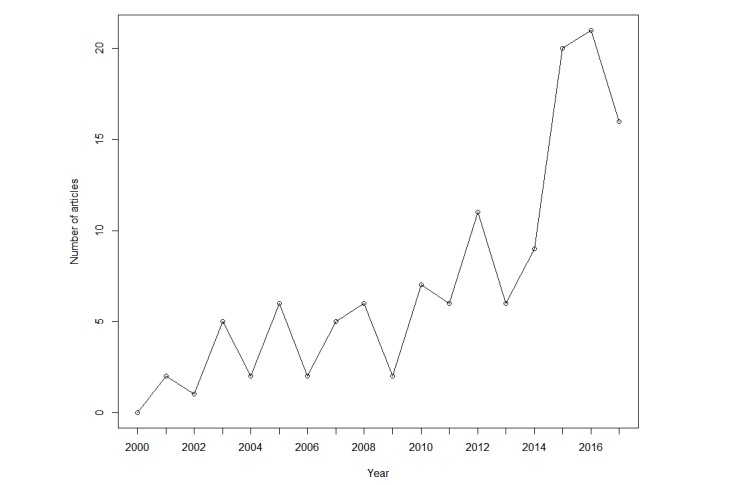
Included articles by year.

**Figure 3:**
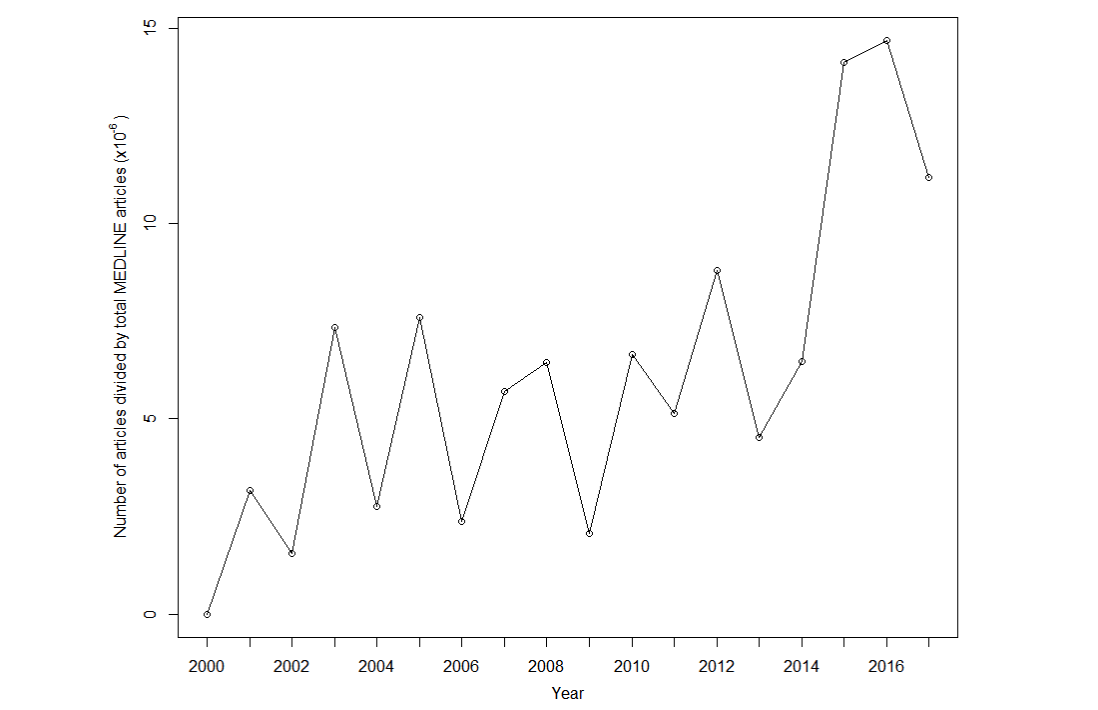
Included articles by year, divided by total number of MEDLINE articles published during that same year.

There was an increase over time (2000-2018) in the prevalence of included articles. The graph showing the number of included articles per year is given in Figure 2. As the amount of total published research has also increased over this same time span, we provide in Figure 3, a graph relative to the total amount of published research in MEDLINE. Each data point is calculated by dividing the number of included articles from a given year by the number of total MEDLINE articles published during that same year (based on MEDLINE’s “Publication Date” field).

### Characteristics of Included Studies

Of the included studies, 40.2% (51/127) were RCTs; 56.7% (72/127) implemented nonrandomized interventions (eg, nonrandomized multiarm studies, single-arm studies, pilot feasibility studies, comparison of multiple measurement methods in 1 group, etc). The remaining 4 [36-39] were surveys or focus groups of potential participants evaluating participant willingness to use specific interventions.

**Figure 4:**
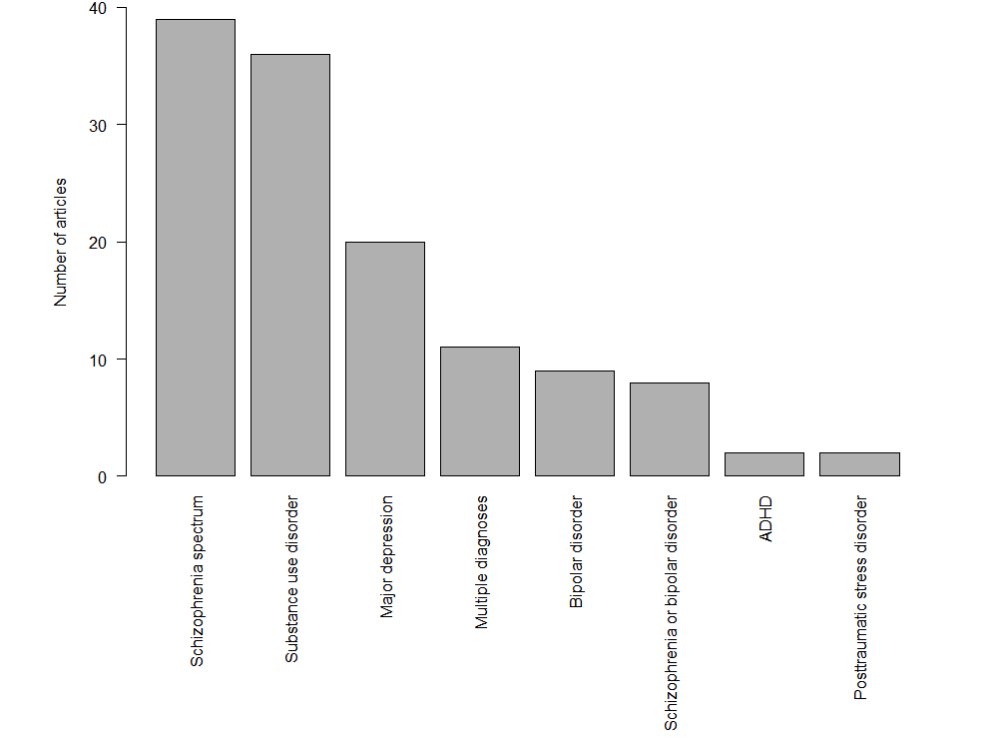
Number of articles by diagnosis. ADHD: attention deficit hyperactivity disorder.

Many psychiatric populations were studied (Figure 4): primary psychotic disorders (n=39), substance use disorders (n=36), major depression (n=20), bipolar disorder (n=9), attention deficit hyperactivity disorder (ADHD; n=2), and PTSD (n=2). Common transdiagnostic populations included “schizophrenia or bipolar disorder” (n=8), dual diagnosis (substance use disorder comorbid with a primary psychiatric disorder, n=3), and pandiagnostic, that is, any psychiatric disorder (n=6). The remaining studies evaluated all patients taking aripiprazole [40] or pooled data from multiple clinical trials of medications for schizophrenia and ADHD [41]. Among the substance use disorders, tobacco was studied most (n=15), followed by opioids (n=11), alcohol (n=5), cannabis (n=2), amphetamines (n=2), and all substance use disorders (n=1).

Studied outcomes included intervention feasibility (n=29), system usability (n=12), and participant satisfaction or acceptability (n=38). Furthermore, 94 studies included some measure of medication adherence, and 32 evaluated or compared the accuracy of one or more medication adherence measurement methods. Many studies evaluated other outcomes, including change in symptoms over time, reduction in substance use, or physiologic metrics such as sleep duration or step count.

### Synthesis

**Figure 5:**
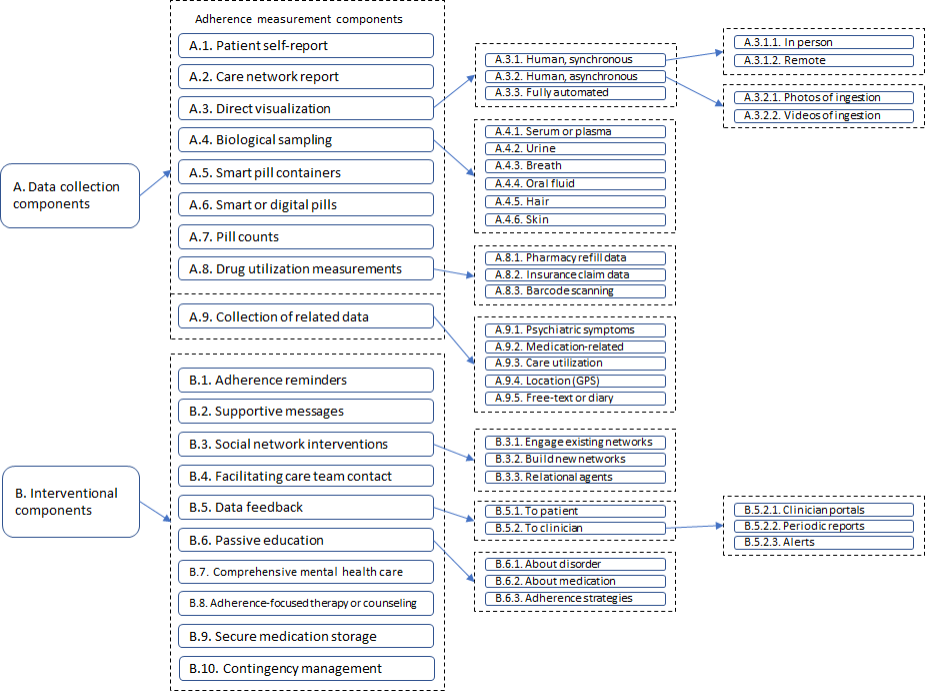
Visual concept map of components of studied technological systems. GPS: global positioning system.

On the basis of the included articles, we constructed a taxonomy of components of the studied technological systems, broken down into the 2 major categories of “data collection components” and “interventional components.” Note that any particular study or technological system may include components from one or both categories. A concept map of the full taxonomy is provided in Figure 5.

### A. Data Collection Components

Adherence measurement and monitoring are critical components of adherence enhancement in many ways, from accurately evaluating the efficacy of interventions designed to improve adherence to identifying patients who would benefit from adherence interventions. The majority of studies collected only adherence data, however, we also included a collection of *related* data within this category (A.9.). This reflects the group of technological systems that collect data other than adherence for downstream use in interventional components.

#### A.1. Patient Self-Report

By far, the most commonly studied method (n=54) for measuring medication adherence was patient self-report. Many multicomponent apps or websites included daily or weekly surveys filled out by the participants; self-report of medication adherence was frequently included in these surveys [42-48]; IVR, telephone, text messaging, and videoconferencing interventions similarly included questions evaluating medication adherence [49-55]. Furthermore, self-report was frequently used as a baseline adherence assessment method with which other, more objective measures were compared [56-59].

#### A.2. Care Network Report

Some studies evaluated the subjective overall adherence report by individuals other than the participant (eg, treating clinicians or participant’s friends, family, or other social support). These reports were not based on direct observation, but rather on subjective, retrospective assessments of a patient’s adherence history or an overall perception of their likelihood to be adherent. This method was rarely used; none of the included studies included it as part of an adherence intervention. However, 6 studies evaluated its accuracy compared with other measurement methods [56-58,60-62]. All 6 studies evaluated 1-time subjective clinician rating scales, and 2 also evaluated reports from a “relative” or a “close informant” [58,62].

#### A.3. Direct Visualization of Medication Ingestion

Many interventions attempted to confirm adherence by *directly visualizing* the process of medication administration*.* Although there were many types of direct visualization methods, all of them involved observation of the patient during the process of physically ingesting the medication.

##### A.3.1: Human Observation, Synchronous

Direct visualization of medication ingestion in real time by a human observer was studied in 4 interventions. A total of 2 studies used direct in-person observation as a gold standard to evaluate the accuracy of other measurement systems (A.3.1.1) [63,64], whereas 2 other studies used mobile videoconferencing technology to observe dosing remotely (A.3.1.2) [65,66]. Both of these studies had a standardized protocol for observed dosing, ostensibly to ensure adherence; the participants kept the pill container on camera while removing the medication and showed their open mouth following pill ingestion to prevent hiding the medication in the cheek.

##### A.3.2. Human Observation, Asynchronous

A related method of visual adherence measurement, studied by 4 included articles, involved the participant capturing static photos or videos of the medication dosing process, to be evaluated in nonreal time (n=4). One study [38] evaluated the acceptability of static photos of pill counts (A.3.2.1) among a population of patients on buprenorphine. A total of 2 other studies discussed daily photographs of individual medication doses taken at the time of ingestion, either as the only intervention [67] or as part of a multicomponent intervention app [68]. Moreover, 1 study discussed nonreal-time video footage (A.3.2.2) captured on mobile phones as an adherence measurement method [69].

##### A.3.3. Computer Observation (Fully Automated)

Overall, 2 included studies evaluated the feasibility, usability, and measurement efficacy of AICure (AICure, LLC), an automated form of direct observation using CV algorithms to confirm pill ingestion in real time without a human observer [41,64]. The platform worked by automatically identifying the patient and drug in real-time video footage taken on mobile devices to confirm ingestion.

#### A.4. Biomarkers and Metabolites

In current clinical practice, measurements of concentrations of medications or medication metabolites in body fluids such as urine or serum are frequently used to assess compliance with the medication. A total of 15 of the included studies either included such measurements as part of a multicomponent intervention [70,71], used serum (A.4.1) or urine (A.4.2) measurements as a gold standard comparator for other adherence measurement approaches [58,64,72], or evaluated a novel system for biological confirmation of adherence. Such systems included measurements from breath (A.4.3) [73,74], oral fluid (A.4.4) [75,76], hair (A.4.5) [77-79], and skin (A.4.6) [80]. Overall, 1 study evaluated the accuracy of urine systems for an experimental cannabinoid [81]. Another study proposed a system of quantitative serum drug monitoring for all psychiatric medications to track adherence and better achieve therapeutic levels [82].

#### A.5. Smart Pill Containers

“Smart” pill containers using digital information technology to measure adherence (eg, Medication Event Monitoring System [MEMS]) were well-represented among the included studies. Most of these systems used electronic components in the cap to detect the opening of the pill bottle. Data were either wirelessly transmitted to a central server periodically or were retrieved from the bottle at clinic visits. A total of 34 studies directly used such a pill container as part of an intervention or evaluated the accuracy of adherence measurement through such a system [56-62,67,70,71,83-106]. The MEMS was by far the most widely used, but variations on it, including a nasal spray monitor [86], were also used. A total of 19 included studies examined antipsychotic medication adherence, 10 studied substance use disorders, and the remaining 3 studied mood disorders. Many more studies used the MEMS as the endpoint measure for an adherence-enhancing intervention.

#### A.6. “Smart” or Digital Pill

A total of 5 studies evaluated the efficacy of a “digital pill” or “digital medicine system” which, upon gastric activation, transmitted a signal to a wearable sensor which then uploaded data to a central server. All of the studies were conducted with antipsychotics meant to be used long term [40,63,107-109].

#### A.7. Pill Counts

One common low-cost method of medication adherence measurement involved single-time counting of pills remaining in a container, either in-person or remotely over videoconferencing software. Pill counts were used (n=12) either as one component of a multicomponent adherence-enhancing intervention or in comparisons of adherence measurement methods [56-59,66,67,70,71,89,96,104,110]. We did not include studies that merely used pill counts to evaluate a study endpoint.

#### A.8. Drug Utilization Measurements

Finally, objective measurements such as pharmacy refills (A.8.1) or insurance claims (A.8.2), which do not directly capture medication administration data but may provide indirect indices of medication possession and adherence, have been used to assess adherence behaviors over the long term (n=3). Overall, 2 studies calculated medication possession ratios and used them in feedback interventions to enhance as well as measure adherence: one with antipsychotics [111] and one with PTSD medications [112]. Another study incorporated barcodes attached to pill containers (A.8.3). Participants scanned these codes with their mobile devices upon receiving the pill containers, allowing for another form of pickup-level verification [68]. Pill count (eg, observing how many pills were left in the container) was another commonly (n=12) used component either in multicomponent adherence-enhancing interventions or in comparisons of adherence-monitoring methods.

#### A.9. Collection of Adherence-Related Data

It was common (n=46) for interventions to collect and track adherence-related data other than medication adherence itself (eg, symptoms and health care utilization) for a variety of downstream uses. As with adherence measurement, we excluded interventions that tracked other data for the purposes of evaluating study endpoints but did not *use* the data in the intervention itself. Data collection modalities included mobile apps, Web forms, phone calls, IVR, one-way text messages, and smart pill dispensers [90]. Most of the data were used in clinician monitoring or alert systems, but some were used to tailor patient-specific motivational therapy (eg, in handling specific drug use triggers in substance use disorders) [113,114]. By far, the most commonly collected data were basic psychiatric symptom assessments (A.9.1), for example, early warning signs of psychosis [105], illicit drug cravings [115], withdrawal symptoms [70,90], mood [107,116], or PTSD symptoms [112]. Other studies tracked attitudes toward medication [117] or medication side effects [118,119] to guide motivational interventions (A.9.2). Others tracked potential triggers or indices for relapse likelihood including stress, anxiety, sleep, and social support [42,44,120]. Some tracked health care utilization metrics, such as primary care visits attended, specialty care referrals followed, and medication changes made (A.9.3) [121,122]. A total of 3 studies [42,116,123] collected real-time global positioning system information through mobile phones (A.9.4); in one of the studies, redirection alerts were provided when patients entered previously assigned high-risk areas [123]. Some studies allowed for free text entry by the participant in the form of online journals which could later be viewed and correlated with adherence and symptomatic data (A.9.5) [42,47].

### B. Adherence-Enhancing Interventions

Any technological component not designed directly to improve medication adherence monitoring accuracy was classified as an “adherence-enhancing” intervention. Generally speaking, these components were designed to enhance a participant’s motivation (eg, motivational intervention or psychoeducation) or ability (eg, reminders or social support engagement) to adhere to pharmacologic therapy.

#### B.1. Reminders

Reminders directed participants to take their medication as prescribed (n=25). Most of these studies (n=14) used short messaging service (SMS) messages sent to a participant’s mobile phone [36,37,39,54,87,124-132]. Usually, these were prespecified reminder messages programmed to be sent at specific times, but some studies examined the use of personalized SMS reminder messages sent by another person, either a “treatment partner” from the community [36] or a social worker [54]. Other messages included reminders delivered as programmatic app notifications, which functioned similarly to SMS reminders [43,52,68,91,118] and often comprised one aspect of multicomponent app interventions. Still other interventions used smart pill containers as the vehicle for reminder delivery—one smart dispenser uses visual and sound alarms to alert the participant when it is time to take medication [95,98], and another similarly uses audio alarms which can be silenced by pressing a button on the pill dispenser [96,105]. Email [45] and an unspecified social media reminder [133] have also been used.

#### B.2. Supportive Messages

Some interventions (n=21) included supportive one-way messages delivered through SMS, IVR, telephone call, or mobile app, which encouraged or motivated the participant. Some used inspirational quotes such as “The journey of a thousand miles starts with a single step” [49], whereas others displayed the length of successful adherence [68], encouraged continued engagement with the platform [113], or provided encouraging health facts, for example, “Today your blood pressure has been reduced to that of a nonsmoker” [134]. Most were generated and sent or displayed automatically [37,114,123,127, 133,135-137], but others were sent as part of a standardized protocol by care team members [53] or lay health support persons [138], or in response to specific behaviors such as persistently elevated adherence [54,118,139] or positive responses to therapeutic questions [55]. Automated relational agents performed this behavior as part of a larger conversation [140,141]. One focus-grouping study found that participants would like to receive personalized self-efficacy messages [39].

#### B.3. Social Enhancement Interventions

This general category focused on leveraging existing social support or building new social support networks for participants.

##### B.3.1. Engaging Existing Social Support

Technological interventions focused on engaging patients’ social support systems (friends, family, etc) in their care were prevalent (n=9). One study focus-grouped an intervention involving a lay “treatment partner” sending SMS antipsychotic adherence reminders to keep the participant engaged in care [36] *.* Another study used an online social network intervention to allow former smokers to support current smokers in adhering to their nicotine replacement therapy and promoting cessation [142]. An automated “conversational agent” encouraged participants in a study of individuals with schizophrenia to recruit a specific member of their social network to provide reminders and logistical support (eg, transportation problems) and later referenced this social support in future conversations [140], whereas a telephone and Web-based intervention encouraged participants to find and contact social supports [142]. Other interventions incorporated a lay health supporter as a *recipient* of other technological interventions such as psychoeducation [143] or adherence reminder systems and data-gathering surveys [125]. Still others [125,138,144,145] looped social support into the intervention by sending them automated periodic progress reports on adherence and symptomatic control and encouraging the social support to reach out to the participant depending on the gathered data. One study allowed participants to choose weekly support phone calls with a family member or friend [145] versus a trained peer support specialist and compared outcomes.

##### B.3.2. Building New Social Support

A total of 6 studies focused instead on building new social support structures for participants. All 6 included online discussion forums or groups where participants could discuss a variety of treatment or adherence-related issues [42,47,123,142,146,147], and all were part of multicomponent interventions. One study [142] included a complete social network of former and current smokers with member profiles and private messaging in addition to public forum posts.

##### B.3.3. Relational Agent

A total of 2 studies evaluated the use of “embodied relational agents” as adherence aids [140,141] in schizophrenia treatment. These relational agents were computer-generated animated humanoid figures who interacted through scripted modules and programmed response trees, allowing for limited bidirectional communication with the participant. The included studies that evaluated relational agents used the agents as a general purpose treatment delivery modality, incorporating psychoeducation, adherence measurement, symptom assessment, behavioral counseling, encouragement, and general purpose social support.

#### B.4. Facilitating Care Team Access

Some adherence interventions (n=13), in particular mobile phone and Web apps, included functionality for the participant to initiate contact with a care team representative at any time. Some [118] allowed the sending of prespecified messages regarding side effects or symptoms or had the study staff screen messages meant for the clinical care team [128,129], whereas others allowed for general purpose two-way messaging through SMS, email, or a messaging system designed separately for the app [42,45,53,65,114,139,147]. Another stored care team contact information within a multicomponent app to enable one-button contact, and displayed this button based on certain user responses [68]. One study collecting adherence data via IVR allowed participants to be transferred to a case manager during the IVR call [148], and another evaluating text message content preferences among potential participants receiving buprenorphine maintenance treatment found that they would prefer frequent provider contact to be available [39].

#### B.5. Data Feedback

This component of interventions was designed to present collected data on medication adherence or adherence-related data to users of the system, including participants, clinicians, or both. Participant feedback was designed to enhance self-knowledge and accountability, whereas clinician feedback enabled monitoring of patient adherence, as well as early and targeted intervention in the case of nonadherence or other treatment concerns.

##### B.5.1. Patient-Directed Feedback

Medication adherence feedback directed toward patients was used in 25 studies. This category included any intervention component which enabled the participant to view their own adherence data, either in real time through a mobile phone or website interface or via periodically generated reports. Frequently, this was one part of multicomponent interventions, but some studies evaluated the effect of feedback alone as an adherence-enhancing intervention [84,85,88,102,103]. Other systems included feedback on more than just adherence data (eg, symptomatic progression as assessed by self-report) [44,46,120]. Note that accurate medication adherence feedback rests on the assumption of an accurate measurement method.

##### B.5.2. Clinician-Directed Feedback

Various systems were developed to keep treating clinicians informed about medication adherence and other related data, to enable earlier and more accurate identification of potential issues for intervention. The data given to the clinician was sourced from many of the previously listed medication measurement components (direct visualization, smart pill dispenser, self-report, etc). Multiple studies (n=31) explicitly mentioned such systems [40,42,44-46,49,51,53,63,65,91,93,95,104,105, 107,108,111,112,114,116,118-120,123,125,138,139,144,145,147], which fell under 3 major categories (not mutually exclusive): “portal” real-time monitoring (n=15), periodic reporting (n=9), and alerts (n=16). Real-time monitoring (B.5.2.1) allows the clinician to, at any time they choose, examine their participants’ medication adherence data, almost always through Web portals [40,44-46,63,65,95,104,105,107,108,116,118,120,147]. Periodic reporting (B.5.2.2) involves standard adherence reports sent at regular intervals, most often through emails [49,51,91,93, 111,125,139,144,145]. Alerts (B.5.2.3) are “push” notifications sent to notify clinicians in circumstances that require immediate response, such as suicidal ideation, hospitalization, and life disturbances such as housing eviction, physical side effects (eg, chest pain), or relapse to drug use [42,44,51,53,65,104, 105,108,111,112,114,119,123,138,144,145]. In some studies, patients’ social support as well as the clinical care team was able to receive reports or alerts [53,125,138,144,145].

#### B.6. Passive Education

Educational interventions were present in 39 included studies [42,45,47,51,64,68,70,71,84,88,91,93,102,105,113,114, 118,123,127,130,134,136,137,139-142,146-157]. Modalities included websites, mobile apps, telephone calls, SMS messages, IVR calls, relational agents, and smart pill dispensers. Frequently presented educational topics included the studied disorder (eg, chronicity, symptom management, triggers, and warning signs), the studied medications (eg, purpose, onset of action, and possible side effects), strategies to improve medication adherence (eg, routines and planning for potential barriers), the importance of medication adherence, coping strategies for setbacks, and the importance of social support. In the vast majority of studies, education was one component of a multicomponent intervention.

#### B.7. Comprehensive Mental Health Care

Many studies (n=22) evaluated medication adherence in the context of remotely delivered psychiatric or mental health care—defined for the purposes of this study as when normal components of mental health care visits (cognitive behavioral therapy [CBT], symptomatic check-ins, and medication adherence counseling) were delivered remotely, through phone calls or videoconferences, by members of the care team including psychiatrists, nurses, pharmacists, and specialized coaches. Some of the studies compared medication adherence (and other outcomes) between telecare and care as usual [158-161], whereas others evaluated the effects on adherence of novel standardized intervention protocols delivered over telephone or videoconference [50,53,65,96,112,115,119,122, 127,143,146,149,152,154-157,162]. The content of novel protocols varied widely, but frequently focused on disease or medication education, symptomatic assessment, and motivational enhancement around medication adherence. Overall, 6 explicitly referenced motivational interviewing [65,112,127,146,149,156] and 3 drew from CBT [127,143,149]. Frequency ranged from daily to monthly. Some were part of multicomponent technological interventions [65,96].

#### B.8. Adherence-Targeted Interactive Psychotherapy or Counseling

Some interventions used technology to deliver adherence-focused psychotherapy or counseling, either remotely as part of remote care or as part of a standardized intervention delivered through an app or a website. One major modality which emerged was motivational intervention around medication adherence (n=9). This was used in remote psychiatric care in the aforementioned 6 studies [65,112,127,146,149,156], but it was also used without a human clinician in 3 mobile phone apps or Web-based interventions [42,47,118]. In one study, the patient was queried about their reasons for nonadherence and, based on the reasons, was provided with tailored motivational feedback [118]. In the others, the cost-benefit framework was used within the website to encourage the participant to evaluate strategies, solve problems, and set goals [42,47]. The other major framework which emerged (n=12) was CBT, which is used as a major component of psychotherapy in many of the studied disorders and can help restructure maladaptive thoughts about psychoactive medication. In the included studies, strategies from CBT were used as part of tele-mental health care [127,143,149], as the comparator [84], or as part of Web or mobile phone app–delivered curricula [42,45,47,123, 134,136,137]. Another study used CBT strategies in a tailored text messaging intervention to probe for unhelpful behaviors or thought patterns, using information collected from the patient [55].

#### B.9. Secure Medication Storage

Some studies used a “security” component designed to prevent overuse or diversion, or prevent theft, of medication (n=3). All 3 of these studies were in opioid use disorder populations receiving buprenorphine [65,70,71] and used a secure locked pill dispenser. In 2 studies, doses were only available during a 3-hour window each day and were taken under the supervision of a nurse. In one study, the dispenser released a daily dose of medication via an access code transmitted during a remote check-in with a mobile recovery coach [65].

#### B.10. Contingency Management

Finally, some studies (n=6) included a contingency management or incentive system which involved prizes or rewards [67-69, 93,118,125]. We did not count studies as having a “contingency management” component if the payment or rewards were given merely for study participation; the incentives had to be built into the overall system and specifically incentivize adherence behaviors or comply with adherence-monitoring protocols. Most of these systems took the form of virtual points which were earned based on specific behaviors, tracked through mobile or Web apps, and could be redeemed for cash. In 3 of these, points were earned for scanning a barcode or sending photos or videos for adherence monitoring purposes [67-69], whereas others were earned by completing education materials, responding to messages, or checking in to adherence enhancement systems [93,118,125].

### Multicomponent Interventions

Multicomponent interventions were those that used multiple intervention components in tandem. Below are tables (Tables 1-4) for the 4 most common disorder contexts (substance use disorder, depression, schizophrenia spectrum, and bipolar disorder) which show the multicomponent apps with at least 4 separate components. We use our framework to categorize and decompose the interventions based on which components they include.

**Table 1:** Comparison of multicomponent interventions for schizophrenia spectrum disorders with 4 or more components, by presence or absence of particular components.

Source	System components^a^												
	A.1-8	A.9	B.1	B.2	B.3	B.4	B.5.1	B.5.2	B.6	B.7	B.8	B.9	B.10
Aschbrenner (2016) [54]	✓^b^	✓	✓	✓	—^c^	—	—	—	—	—	—	—	—
Beebe (2014) [50]	✓	✓	—	—	—	—	—	—	—	✓	✓	—	—
Ben-Zeev (2014) [139]	✓	—	—	✓	—	✓	—	✓	✓	—	—	—	—
Bickmore (2010) [140]	✓	—	—	✓	✓	—	✓	—	✓	—	—	—	—
Granholm (2012) [55]	✓	✓	—	✓	—	—	—	—	—	—	✓	—	—
Kane (2013) [63]	✓	✓	—	—	—	—	✓	✓	—	—	—	—	—
Kreyenbuhl (2016) [12]	✓	✓	✓	✓	—	✓	✓	✓	✓	—	✓	—	✓
Ruskin (2003) [105]	✓	✓	✓	—	—	—	—	✓	✓	—	—	—	—
Stentzel (2016) [53]	✓	✓	—	✓	—	✓	—	✓	—	✓	—	—	—
Xu (2016) [125]	—	✓	✓	—	✓	—	—	✓	—	—	—	—	✓

^a^A.1-8: Adherence data collection. A.9: Collection of related data. B.1: Adherence reminders. B.2: Supportive messages. B.3: Social network interventions. B.4: Facilitating care team contact. B.5.1: Data feedback to patient. B.5.2: Data feedback to clinician. B.6: Education. B.7: Comprehensive mental health care. B.8: Adherence-targeted psychotherapy. B.9: Secure medication storage. B.10: Contingency management.

^b^✓: Component is present.

^c^—: Component is not present.

**Table 2:** List of multicomponent interventions for bipolar disorder with 4 or more components, by presence or absence of particular components.

Source	System component^a^												
	A.1-8	A.9	B.1	B.2	B.3	B.4	B.5.1	B.5.2	B.6	B.7	B.8	B.9	B.10
Faurholt-Jepsen (2014) [44]	✓^b^	✓	—^c^	—	—	—	✓	✓	—	—	—	—	—
Lauder (2017) [42]	✓	✓	—	—	✓	✓	✓	✓	✓	—	✓	—	—
Sajatovic (2015) [93]	✓	—	—	—	—	—	✓	✓	✓	—	—	—	✓
Wenze (2014) [136]	✓	✓	—	✓	—	—	—	—	✓	—	✓	—	—

^a^A.1-8: Adherence data collection. A.9: Collection of related data. B.1: Adherence reminders. B.2: Supportive messages. B.3: Social network interventions. B.4: Facilitating care team contact. B.5.1: Data feedback to patient. B.5.2: Data feedback to clinician. B.6: Education. B.7: Comprehensive mental health care. B.8: Adherence-targeted psychotherapy. B.9: Secure medication storage. B.10: Contingency management.

^b^✓: component is present.

^c^—: component is not present.

**Table 3:** Comparison of multicomponent interventions for substance use disorder with 4 of more components, by presence or absence of particular components.

Source	System component^a^												
	A.1-8	A.9	B.1	B.2	B.3	B.4	B.5.1	B.5.2	B.6	B.7	B.8	B.9	B.10
Gordon (2017) [68]	✓^b^	✓	✓	✓	—^c^	✓	✓	—	✓	—	—	—	✓
Gustafson (2016) [123]	—	✓	—	✓	✓	—	✓	✓	✓	—	✓	—	—
McClure (2016) [114]	—	✓	—	✓	—	✓	—	✓	✓	—	—	—	—
Mooney (2007) [84]	✓	—	—	—	—	—	✓	—	✓	—	✓	—	—
Schuman-Olivier (2018) [65]	✓	✓	—	—	—	✓	—	✓	—	—	✓	✓	—
Sigmon (2015) [71]	✓	✓	—	—	—	—	—	—	✓	—	—	✓	—
Swan (2012) [146]	—	✓	—	—	✓	—	✓	—	✓	✓	✓	—	—
Tseng (2017) [127]	—	—	✓	✓	—	—	—	—	✓	✓	✓	—	—

^a^A.1-8: Adherence data collection. A.9: Collection of related data. B.1: Adherence reminders. B.2: Supportive messages. B.3: Social network interventions. B.4: Facilitating care team contact. B.5.1: Data feedback to patient. B.5.2: Data feedback to clinician. B.6: Education. B.7: Comprehensive mental health care. B.8: Adherence-targeted psychotherapy. B.9: Secure medication storage. B.10: Contingency management.

^b^✓: component is present

^c^—: component is not present.

**Table 4:** Comparison of multicomponent interventions for major depressive disorder with 4 or more components, by presence or absence of particular components.

Source	System component^a^												
	A.1-8	A.9	B.1	B.2	B.3	B.4	B.5.1	B.5.2	B.6	B.7	B.8	B.9	B.10
Aikens (2015) [138]	✓^b^	✓	—^c^	✓	✓	—	—	✓	—	—	—	—	—
Fortney (2007) [157]	✓	✓	—	—	—	—	—	—	✓	✓	—	—	—
Gervasoni (2010) [119]	✓	✓	—	—	—	—	—	✓	—	✓	✓	—	—
Lauritsen (2017) [46]	✓	✓	—	—	—	—	✓	✓	—	—	—	—	—
Meglic (2012) [147]	—	✓	—	—	✓	✓	✓	✓	✓	—	—	—	—
Mohr (2015) [91]	✓	✓	✓	—	—	—	✓	✓	✓	—	—	—	—
Pfeiffer (2017) [145]	✓	✓	—	—	✓	—	—	✓	—	—	—	—	—
Piette (2013) [144]	✓	✓	—	—	✓	—	—	✓	—	—	—	—	—
Rickles (2005) [152]	✓	✓	—	—	—	—	—	—	✓	✓	—	—	—
Robertson (2006) [45]	✓	✓	✓	—	—	✓	✓	✓	✓	—	✓	—	—
Rusche-Skolarus (2015) [51]	✓	✓	—	—	—	—	—	✓	✓	—	—	—	—

^a^A.1-8: Adherence data collection. A.9: Collection of related data. B.1: Adherence reminders. B.2: Supportive messages. B.3: Social network interventions. B.4: Facilitating care team contact. B.5.1: Data feedback to patient. B.5.2: Data feedback to clinician. B.6: Education. B.7: Comprehensive mental health care. B.8: Adherence-targeted psychotherapy. B.9: Secure medication storage. B.10: Contingency management.

^b^✓: component is present.

^c^—: component is not present.

## Discussion

### Principal Findings

Our study is the first to create a literature-driven taxonomy of the components of adherence-monitoring methods and adherence-enhancing interventions used across adult psychiatric disorders. There is a proliferation of frameworks used to discuss these technological interventions, some of which are borrowed from nonpsychiatric adherence studies. We hope that this study will build on existing frameworks to provide a definitive framework for this specific context; in particular, we hope to capture the breadth of studied interventions, and provide a uniform vocabulary with which to discuss and compare them. This taxonomy may prove useful during the design phase of a new intervention, where investigators or clinicians may choose to include relevant components from the framework to suit their population or outcome of interest.

Although the framework was constructed bottom-up from the literature, it does ultimately appear to parallel existing frameworks of nonadherence behaviors. Reminders to take medication primarily address *involuntary* nonadherence concerns, whereas security and monitoring interventions often address *voluntary* nonadherence [20]. Educational interventions often aim to provide information and correct misconceptions about disorders, medications, and side effects (addressing *perceptual* nonadherence factors), whereas supportive interventions such as social support engagement and online community building attempt to address the well-studied association between perceived social support and medication nonadherence [163]. It is perhaps not surprising that adherence interventions parallel perceived causes of nonadherence, but it is reassuring nonetheless.

Many of the intervention types have been delivered using various *modalities.* It is useful to draw a distinction between the intervention *type* or *component* (eg, educational, reminder, supportive, feedback, etc) and the *modality* by which it is delivered (SMS, IVR, app, website, etc). Although certain modalities of delivering certain interventions may ultimately prove to have advantages over others (for instance, navigating a mobile app may well be more acceptable than waiting for IVR dialogues when filling out an adherence survey), it is not yet clear from the literature whether this is the case. As technology continues to evolve, there will no doubt be new modalities of intervention delivery (for instance, the once prevalent studies of IVR and telephonic interventions have largely been supplanted in recent years by SMS text messaging, website, and mobile phone app studies). One notable exception: the distinction between *modality* and *intervention type* does not apply to most data collection components (smart pill dispensers, body fluid sampling, and digital pills) as the two are inextricably tied.

The body of literature on this topic is growing, as reflected by the increasing number of studies included from recent years. However, looking at the literature from any individual psychiatric or substance use disorder highlights the fact that the field remains young, and most studies conducted are small-n pilot studies. Furthermore, large RCTs are necessary to elucidate the impact of particular interventions.

We found that most components are used transdiagnostically, that is, they have been studied in schizophrenia spectrum disorders, mood disorders, multiple substance use disorders, and PTSD (ie, most psychiatric disorders which have a pharmacologic component to therapy). Data collection components (including smart pill dispensers, periodic self-report, and body fluid testing), reminders, educational interventions, tele-health–driven counseling, and multicomponent disease management apps have been explored in most or all psychiatric disorders. This suggests that despite the differences in the mental state between different disorders, and perhaps even different primary reasons for nonadherence, there is still much to be learned and transferred from one domain to the others. Particularly, when designing a new intervention for clinical or research use, investigators would do well to peruse the literature from different psychiatric disorders for ideas and related work. It is possible that transdiagnostic interventions (perhaps with disorder-specific education or support modules) might ultimately improve medication adherence in a general psychiatric population. However, as of yet, very little transdiagnostic research has been conducted in this field.

Multicomponent interventions, including comprehensive disease management apps, have been studied in the majority of psychiatric disorders. These interventions present a challenge from a research standpoint, as it is difficult to assess the impact of any individual component without large, factorial-design studies. However, there may be synergistic adherence effects from comprehensive disease management apps, in the same way that standard psychiatric care visits address a wide variety of adherence concerns and motivational factors. Furthermore, if these interventions are to one day be used regularly in clinical practice, multicomponent interventions are more practical from an implementation standpoint.

One surprising fact is that many studies which evaluated the effect of adherence-enhancing interventions used *self-reported* adherence, or adherence as measured by a smart pill dispenser, as the endpoint. Studies have shown that self-reported adherence frequently overestimates true adherence [58,65,164,165]; this fact may compromise or blur results from many of these studies. Biological measurements and directly observed ingestion provide far better adherence assessment, but are costly, inconvenient, and often impractical to implement. Novel adherence measurement systems, such as digital pills, automated adherence monitoring, and remote direct observation, may ultimately provide a better compromise between accuracy and practicality in future trials. In fact, some of these novel methods have been evaluated and used in clinical trials already. Accurate assessment of adherence is crucial to evaluating the efficacy of any of these interventions, and future investigators would do well to select their adherence measurement endpoint wisely.

### Strengths and Limitations

The strengths of our study include the breadth of the topic area and the comprehensive coverage of all types of interventions, as well as the literature-guided taxonomic framework. Our tables of multicomponent interventions further show the utility of this transdiagnostic framework to decompose and categorize interventions across all adult mental health and substance use disorders. However, our study is limited by this same breadth; we were unable to conduct a quantitative synthesis or formal meta-analysis of all included studies given the wide variety of interventions, populations, and outcomes. In the future, we plan to build off of this study and conduct more focused comparisons of specific topic areas.

### Conclusions

In conclusion, we provide a systematic review of the literature on technological interventions for medication adherence and monitoring in mental health and substance use disorders; we then use the included studies to develop a literature-driven transdiagnostic framework of intervention components. We hope this will help guide future research in the field and ultimately lead to further clinical applications.

## Appendix

Multimedia Appendix 1 Search strategies for included databases.

Multimedia Appendix 2 Details of included studies.

## Abbreviations

AI: artificial intelligence
ADHD: attention deficit hyperactivity disorder
CBT: cognitive behavioral therapy
CENTRAL: Cochrane Central Register of Controlled Trials
CV: computer vision
IVR: interactive voice response
MEMS: Medication Event Monitoring System
PTSD: posttraumatic stress disorder
RCTs: randomized controlled trials
SMS: short messaging service
