# Supplementary material for: Technological Interventions for Medication Adherence in Adult Mental Health and Substance Use Disorders: A Systematic Review
Source: JMIR Ment Health. 2019 Mar 12;6(3):e12493. doi: 10.2196/12493 (PMC6434404; doi:10.2196/12493)
Supplement: Multimedia Appendix 1 [file mental_v6i3e12493_app1.pdf]

Appendix 1: Search Strategy
MEDLINE (PubMed)

((“Technology”[Mesh] OR technolog*[tiab])

OR

(“Artificial Intelligence” [Mesh] OR “Artificial Intelligence”[tiab] OR “AI”[tiab] OR “computational intelligence”[tiab] OR “machine intelligence”[tiab] OR “computer vision”[tiab])

OR

(“Telemedicine”[Mesh] OR “mobile health”[tiab] OR “mhealth”[tiab] OR “eHealth”[tiab] OR “digital health”[tiab] OR “digital medicine”[tiab] OR “telehealth”[tiab])

OR

(“Cell Phone”[Mesh] OR cell phone*[tiab] OR cellular phone*[tiab] OR cell telephone*[tiab] OR cellular telephone*[tiab] OR portable cellular phone*[tiab] OR transportable cellular phone*[tiab] OR mobile phone*[tiab] OR mobile telephone*[tiab] OR smartphone*[tiab] OR smart phone*[tiab] OR text messag*[tiab] OR “texting”[tiab] OR “short message service”[tiab] OR “sms”[tiab])

OR

(“Internet”[Mesh] OR internet*[tiab] OR “world wide web”[tiab] OR website*[tiab] OR web site*[tiab])

OR

(“Wireless Technology”[Mesh] OR wireless technolog*[tiab])

OR

(“Monitoring, Ambulatory”[Mesh] OR “ambulatory monitoring”[tiab] OR “monitoring”[tiab])

OR

(“Videoconferencing”[Mesh] OR videoconferenc*[tiab])

OR

(“ingestible sensor”[tiab] OR ingest*[tiab] AND sensor*[tiab])

OR

(“biomarker”[tiab] OR “biomarkers”[Mesh])

OR

(“Software”[Mesh] OR “software”[tiab] OR computer program*[tiab] OR software tool*[tiab] OR “software engineering”[tiab] OR computer application*[tiab] OR mobile application*[tiab] OR mobile app*[tiab] OR “app”[tiab] or “apps”[tiab]))

AND

((“Medication Adherence”[Mesh] OR “Patient Compliance”[Mesh] OR medication adheren*[tiab] OR medication complian*[tiab] OR adheren*[tiab] OR complian*[tiab] OR nonadheren*[tiab] OR noncomplian*[tiab]))

AND

((“Psychiatry”[Mesh] OR “psychiatr*”[tiab])

OR

(“Mental health”[Mesh] OR “mental health”[tiab])

OR

(“Behavioral Medicine”[Mesh] OR “behavioral health”[tiab] OR “behavioral medicine”[tiab])

OR

("Substance-Related Disorders"[Mesh] OR “substance-related disorder*”[tiab] OR “drug dependence”[tiab] OR “drug addiction”[tiab] OR “substance use disorder*”[tiab] OR “substance abuse”[tiab] OR “substance dependence”[tiab] OR “drug use disorder*”[tiab] OR “controlled substances”[Mesh] OR “controlled substance*”[tiab])

OR

(“narcotic”[Mesh] OR “narcotic*”[tiab] OR “opioid*”[tiab] OR “opiate*”[tiab] OR “buprenorphine”[mesh] OR “buprenorphine”[tiab] OR “methadone”[mesh] OR “methadone”[tiab])

OR

(“ethanol”[Mesh] OR “ethanol”[tiab] OR “alcohol*”[tiab])

OR

(“tobacco use”[Mesh] OR “tobacco products”[Mesh] OR “tobacco”[tiab] OR “cigarette*”[tiab])

OR

(“amphetamine”[mesh] OR “amphetamine*”[tiab] OR “methamphetamine*”[tiab])

OR

(“cocaine”[mesh] OR “cocaine”[tiab])

OR

(“cannabis”[mesh] OR “cannabis”[tiab] OR “marijuana”[tiab])

OR
("Sleep Disorders, Intrinsic"[Mesh] OR “insomnia”[tiab] OR “narcolepsy”[tiab])

OR

("Stress Disorders, Post-Traumatic"[Mesh] OR “PTSD”[tiab] OR “post-traumatic stress disorder”[tiab])

OR

(“Depression”[Mesh] OR “depressive disorder”[Mesh] OR “depress*”[tiab])

OR

(“bipolar disorder”[Mesh] OR “bipolar disorder”[tiab] OR “bipolar depression”[tiab] OR “manic”[tiab] OR “mania”[tiab])

OR

(“anxiety disorders”[mesh] OR “anxiety disorder*”[tiab] OR “Anxiety”[Mesh] OR anxi*[tiab] OR “Anti-Anxiety Agents”[Mesh] OR “social anxiety”[tiab] OR “Panic”[Mesh] OR “Panic Disorder”[Mesh] OR panic[tiab] OR “Phobic Disorders”[Mesh] OR phobi*[tiab])

OR

(“Personality Disorders”[Mesh] OR personality disorder*[tiab])

OR

((“Attention Deficit Disorder with Hyperactivity”[Mesh] OR “Central Nervous System Stimulants”[Mesh] OR stimulant*[tiab] OR "ADHD"[tiab] OR "attention deficit"[tiab] OR “ADD”[tiab]) AND ("Adult"[Mesh] OR adult*[tiab])))

OR

("Schizophrenia Spectrum and Other Psychotic Disorders"[Mesh] OR “schizophrenia”[tiab] OR “psychosis”[tiab] OR “psychotic”[tiab])

OR

(“chronic pain”[Mesh] OR “chronic pain”[tiab])

OR

(OR “obsessive-compulsive disorder”[tiab] OR “OCD”[tiab])

OR

(“feeding and eating disorders”[mesh] OR “eating disorder*”[tiab] OR “feeding disorder*”[tiab] OR “bulimia”[tiab] OR “binge-eating”[tiab])

OR

(“mental disorders”[mesh:NoExp]))

AND

("2000"[Date - Publication] : "3000"[Date - Publication])

EMBASE

('technology'/exp OR 'technology':ti,ab OR 'artificial intelligence'/exp OR 'artificial intelligence':ti,ab OR 'ai':ti,ab OR 'computer intelligence':ti,ab OR 'machine intelligence':ti,ab OR 'computer vision':ti,ab OR 'telemedicine'/exp OR 'telemedicine':ti,ab OR 'mobile health'/exp OR 'mobile health':ti,ab OR 'mhealth':ti,ab OR 'ehealth':ti,ab OR 'digital health':ti,ab OR 'digital medicine':ti,ab OR 'telehealth':ti,ab OR 'mobile phone'/exp OR 'cell phone*':ti,ab OR 'cellular phone*':ti,ab OR 'mobile phone*':ti,ab OR 'mobile telephone*':ti,ab OR 'smartphone*':ti,ab OR 'smart phone*':ti,ab OR 'text messaging'/exp OR 'text messag*':ti,ab OR 'short message service':ti,ab OR 'sms':ti,ab OR 'internet'/exp OR 'internet':ti,ab OR 'world wide web':ti,ab OR 'website*':ti,ab OR 'web site':ti,ab OR 'web site'/exp OR 'wireless communication'/exp OR 'wireless communication':ti,ab OR 'wireless technology':ti,ab OR 'monitoring'/exp OR 'monitoring':ti,ab OR 'videoconferencing'/exp OR 'videoconferenc*':ti,ab OR ‘biomarker’/exp OR ‘biomarker’:ti,ab OR 'software'/exp OR 'mobile application'/exp OR 'software':ti,ab OR 'computer program*':ti,ab OR 'software engineering':ti,ab OR 'computer application*':ti,ab OR 'mobile application*':ti,ab OR 'mobile app*':ti,ab OR 'app':ti,ab OR 'apps':ti,ab) AND ('medication compliance'/exp OR 'patient compliance'/exp OR 'complianc*':ti,ab OR 'noncomplian*':ti,ab OR 'adheren*':ti,ab OR 'nonadheren*':ti,ab) AND ('psychiatry'/exp OR 'psychiatr*':ti,ab OR 'mental health'/exp OR 'mental health':ti,ab OR 'behavioral health'/exp OR 'behavioral health':ti,ab OR 'behavioral medicine':ti,ab OR 'substance use'/exp OR 'drug dependence'/exp OR 'drug use'/exp OR 'substance-related disorder*':ti,ab OR 'drug dependence':ti,ab OR 'drug addiction':ti,ab OR 'substance use disorder*':ti,ab OR 'substance abuse':ti,ab OR 'substance dependence':ti,ab OR 'drug use disorder*':ti,ab OR 'controlled substance*':ti,ab OR 'narcotic analgesic agent'/exp OR 'narcotic*':ti,ab OR 'opioid*':ti,ab OR 'opiate*':ti,ab OR 'buprenorphine':ti,ab OR 'methadone':ti,ab OR 'naltrexone':ti,ab OR 'naltrexone'/exp OR 'buprenorphine'/exp OR 'buprenorphine plus naloxone'/exp OR 'methadone'/exp OR 'alcohol*':ti,ab OR 'ethanol':ti,ab OR 'alcohol'/exp OR 'alcoholism'/exp OR 'tobacco use'/exp OR 'tobacco'/exp OR 'smoking'/exp OR 'smoking cessation'/exp OR 'tobacco':ti,ab OR 'smoking':ti,ab OR 'cigarette':ti,ab OR 'amphetamine'/exp OR 'amphetamine*':ti,ab OR 'methamphetamine*':ti,ab OR 'cocaine':ti,ab OR 'cocaine dependence'/exp OR 'cocaine'/exp OR 'cannabis'/exp OR 'cannabis addiction'/exp OR 'cannabis':ti,ab OR 'marijuana':ti,ab OR 'sleep disorder'/exp OR 'insomnia':ti,ab OR 'narcolepsy':ti,ab OR 'posttraumatic stress disorder'/exp OR 'ptsd':ti,ab OR 'posttraumatic stress disorder':ti,ab OR 'depress*':ti,ab OR 'mood disorder'/exp OR 'bipolar':ti,ab OR 'manic':ti,ab OR 'mania':ti,ab OR 'psychosis'/exp OR 'schizophrenia spectrum disorder'/exp OR 'schizophreni*':ti,ab OR

'psychosis':ti,ab OR 'psychotic':ti,ab OR 'chronic pain'/exp OR 'chronic pain':ti,ab OR 'mental disease'/de OR 'anxiety disorder'/exp OR 'anxiety disorder*':ti,ab OR ‘social anxiety’:ti,ab OR 'panic'/exp OR 'panic':ti,ab OR 'phobia'/exp OR 'phobi*':ti,ab OR 'personality disorder'/exp OR 'personality disorder':ti,ab OR ‘attention deficit’:ti,ab OR 'ADHD':ti,ab OR 'attention deficit disorder'/exp OR 'ADD':ti,ab OR 'central stimulant agent'/exp OR 'obsessive-compulsive disorder':ti,ab OR 'ocd':ti,ab OR 'eating disorder'/exp OR 'bulimia':ti,ab OR 'binge-eating':ti,ab) AND [adult]/lim AND [humans]/lim AND [english]/lim AND [2000-2017]/py

Web of Science

TI=((technolog* OR "artificial intelligence" OR AI OR "computational intelligence" OR "machine intelligence" OR "computer vision" OR telemedicine OR "mobile health" OR mhealth OR ehealth OR "digital health" OR "digital medicine" OR telehealth OR "cell phone*" OR "cellular phone*" OR "cell telephone*" OR "portable cellular phone*" OR "transportable cellular phone*" OR "mobile phone*" OR "mobile telephone*" OR smartphone* OR "smart phone*" OR "text messag*" OR texting OR "short message service" OR sms OR Internet OR "world wide web" OR "web site*" OR website* OR "wireless technolog*" OR monitoring OR videoconference* OR software OR "computer program*" OR "software tool*" OR "software engineering" OR "computer application*" OR "mobile application*" OR "mobile app*" OR app OR apps) AND (adheren* OR complian* OR noncomplian* OR nonadheren* OR "non-adheren*") AND (psychiatr* OR "mental health" OR "behavioral health" OR "behavioral medicine" OR "drug dependence" OR "drug addiction" OR "substance use" OR "substance abuse" OR "substance dependence" OR "drug use" OR "drug abuse" OR "controlled substance*" OR narcotic* OR opioid* OR opiate* OR buprenorphine OR methadone OR ethanol OR alcohol* OR tobacco OR cigarette OR amphetamine OR methamphetamine OR cocaine OR cannabis OR marijuana OR "sleep disorder*" OR insomnia OR narcolepsy OR PTSD OR "posttraumatic stress disorder" OR "post-traumatic stress disorder" OR depress* OR "mood disorder*" OR bipolar OR manic OR mania OR schizophreni* OR psychosis OR psychotic OR "chronic pain" OR anxiety OR ‘social anxiety’ OR panic OR phobi* OR 'personality disorder*' OR ADHD OR 'attention deficit disorder' OR “ADD” OR “stimulant*” OR "obsessive-compulsive disorder" OR OCD OR "eating disorder" OR bulimia OR "binge eating"))) OR (TS=((technolog* OR "artificial intelligence" OR AI OR "computational intelligence" OR "machine intelligence" OR "computer vision" OR telemedicine OR "mobile health" OR mhealth OR ehealth OR "digital health" OR "digital medicine" OR telehealth OR "cell phone*" OR "cellular phone*" OR "cell telephone*" OR "portable cellular phone*" OR "transportable cellular phone*" OR "mobile phone*" OR "mobile telephone*" OR smartphone* OR "smart phone*" OR "text messag*" OR texting OR "short message service" OR sms OR Internet OR "world wide web" OR "web site*" OR website* OR "wireless technolog*" OR monitoring OR videoconference* OR biomarker* OR “ingestible sensor” OR software OR "computer program*" OR "software tool*" OR "software engineering" OR "computer application*" OR "mobile application*" OR "mobile app*" OR app OR apps) AND (adheren* OR complian* OR noncomplian* OR nonadheren* OR "non-adheren*") AND (psychiatr* OR "mental health" OR "behavioral health" OR "behavioral medicine" OR "drug dependence" OR "drug addiction" OR "substance use" OR "substance abuse" OR "substance dependence" OR "drug use" OR "drug abuse" OR "controlled substance*" OR narcotic* OR opioid* OR opiate* OR buprenorphine OR methadone OR ethanol OR alcohol* OR tobacco OR cigarette OR amphetamine OR methamphetamine OR cocaine OR cannabis OR marijuana OR "sleep disorder*" OR insomnia OR narcolepsy OR PTSD OR "posttraumatic stress disorder" OR "post-traumatic stress disorder" OR depress* OR "mood disorder*" OR bipolar OR manic OR mania OR schizophreni* OR psychosis OR psychotic OR "chronic pain" OR anxiety OR ‘social anxiety’ OR panic OR phobi* OR 'personality disorder*' OR ADHD OR 'attention deficit disorder' OR “ADD” OR “stimulant*” OR "obsessive-compulsive disorder" OR OCD OR "eating disorder" OR bulimia OR "binge eating"))

LANGUAGE: (English)

DOCUMENT TYPES: (Article)

PsycINFO

(technolog* OR artificial intelligence OR ai OR computational intelligence OR machine intelligence OR computer vision OR telemedicine OR mobile health OR mhealth OR ehealth OR digital health OR digital medicine OR telehealth OR cell phone* OR cellular phone* OR cell telephone* OR portable cellular phone* OR transportable cellular phone* OR mobile phone* OR mobile telephone* OR smartphone* OR smart phone* OR text messag* OR texting OR short message service OR sms OR Internet OR world wide web OR web site* OR website* OR wireless technolog* OR monitoring OR videoconference* OR biomarker* OR ingestible sensor* OR software OR computer program* OR software tool* OR software engineering OR computer application* OR mobile application* OR mobile app* OR app OR apps) AND (adheren* OR complian* OR noncomplian* OR nonadheren* OR non-adheren*) AND (psychiatr* OR mental health OR behavioral health OR behavioral medicine OR drug dependence OR drug addiction OR substance use OR substance abuse OR substance dependence OR drug use OR drug abuse OR controlled substance* OR narcotic* OR opioid* OR opiate* OR buprenorphine OR methadone OR ethanol OR alcohol* OR tobacco OR cigarette OR amphetamine OR methamphetamine OR cocaine OR cannabis OR marijuana OR sleep disorder* OR insomnia OR narcolepsy OR ptsd OR posttraumatic stress disorder OR post-traumatic stress disorder OR depress* OR mood disorder* OR bipolar OR manic OR mania OR schizophreni* OR psychosis OR psychotic OR chronic pain OR anxiety OR social anxiety OR panic OR phobi* OR personality disorder OR ADD OR ADHD OR attention deficit OR stimulant OR obsessive-compulsive disorder OR ocd OR eating disorder OR bulimia OR binge eating)

Cochrane CENTRAL

(technolog* OR "artificial intelligence" OR AI OR "computational intelligence" OR "machine intelligence" OR "computer vision" OR telemedicine OR "mobile health" OR mhealth OR ehealth OR "digital health" OR "digital medicine" OR telehealth OR "cell phone*" OR "cellular phone*" OR "cell telephone*" OR "portable cellular phone*" OR "transportable cellular phone*" OR "mobile phone*" OR "mobile telephone*" OR smartphone* OR "smart phone*" OR "text messag*" OR texting OR "short message service" OR sms OR Internet OR "world wide web" OR "web site*" OR website* OR "wireless technolog*" OR monitoring OR videoconference* OR biomarker* OR “ingestible sensor*” OR software OR "computer program*" OR "software tool*" OR "software engineering" OR "computer application*" OR "mobile application*" OR "mobile app*" OR app OR apps) AND (adheren* OR complian* OR noncomplian* OR nonadheren* OR "non-adheren*") AND (psychiatr* OR "mental health" OR "behavioral health" OR "behavioral medicine" OR "drug dependence" OR "drug addiction" OR "substance use" OR "substance abuse" OR "substance dependence" OR "drug use" OR "drug abuse" OR "controlled substance*" OR narcotic* OR opioid* OR opiate* OR buprenorphine OR methadone OR ethanol OR alcohol* OR tobacco OR cigarette OR amphetamine OR methamphetamine OR cocaine OR cannabis OR marijuana OR "sleep disorder*" OR insomnia OR narcolepsy OR PTSD OR "posttraumatic stress disorder" OR "post-traumatic stress disorder" OR depress* OR "mood disorder*" OR bipolar OR manic OR mania OR schizophreni* OR psychosis OR psychotic OR "chronic pain" OR anxiety OR “social anxiety” OR panic OR phobi* OR “personality disorder” OR ADD OR ADHD OR “attention deficit” OR stimulant OR "obsessive-compulsive disorder" OR OCD OR "eating disorder" OR bulimia OR "binge eating")

CLINICALTRIALS.GOV

adherence | compliance | nonadherence | noncompliance

Condition: "alcohol drinking" OR "alcohol-related disorders" OR "alcoholism" OR “attention deficit” OR "amphetamine-related disorders" OR "anhedonia" OR "anxiety disorders" OR "behavior, addictive" OR "bipolar and related disorders" OR "bipolar disorder" OR "compulsive behavior" OR "depression" OR "depressive disorder, major" OR "drinking behavior" OR "dyssomnias" OR "dysthymic disorder" OR "heroin dependence" OR "marijuana abuse" OR "mental disorders" OR "mood disorders" OR "opioid-related disorders" OR "panic disorder" OR “personality disorders” OR “phobic disorder” OR "psychotic disorders" OR "schizophrenia" OR "schizophrenia spectrum and other psychotic disorders" OR "sleep disorders, intrinsic" OR "stress disorders, post-traumatic" OR "stress disorders, traumatic" OR "substance abuse, intravenous" OR "substance-related disorders" OR "tobacco use disorder" OR "trauma and stressor related disorders".

Manually screened for technology

ENGINEERING VILLAGE

((((((((mental* OR $mood OR $psychiatric OR schizophreni* OR $personality OR $bipolar OR depress* OR $anxiet* OR $panic OR $phobi* OR $personality $disorder OR $attention $deficit OR $ADHD OR $stress OR $PTSD OR $abuse OR $abusing OR $abused OR alcohol* OR addiction* OR $smoking OR cigarette* OR $tobacco OR $heroin OR $nicotine OR opioid* OR opiate* OR $methadone OR $substance $abuse OR $substance $use OR $controlled $substance OR $controlled $substances) WN ALL) AND (1896-2018 WN YR)) AND (((((((medication* NEAR/2 adheren*) WN ALL) OR ((medication* NEAR/2 complian*) WN ALL)) OR ((medication* NEAR/2 reminder*) WN ALL)) AND (1896-2018 WN YR)) OR (((((prescription* NEAR/2 adheren*) WN ALL) OR ((prescription* NEAR/2 complian*) WN ALL)) OR ((prescription* NEAR/2 reminder*) WN ALL)) AND (1896-2018 WN YR)) OR ((((($pill NEAR/2 adheren*) WN ALL) OR (($pill NEAR/2 complian*) WN ALL)) OR (($pill NEAR/2 reminder*) WN ALL)) AND (1896-2018 WN YR)))))) AND ((2017 OR 2016 OR 2015 OR 2014 OR 2013 OR 2012 OR 2011 OR 2010 OR 2009 OR 2008 OR 2007 OR 2006 OR 2005 OR 2004 OR 2003 OR 2002 OR 2001 OR 2000) WN YR))
